# Supplementary material for: Flupyradifurone Exhibits Greater Toxicity to the Asian Bumblebee Bombus lantschouensis Compared to the European Bumblebee Bombus terrestris
Source: Insects. 2025 Apr 25;16(5):455. doi: 10.3390/insects16050455 (PMC12112515; doi:10.3390/insects16050455)
Supplement: Supplementary file 1 [file insects-16-00455-s001.zip › insects-3529929-supplementary.pdf]

Table S1. Screening and first-tier step assessment calculations for spray application.

Type of assessment	Screening step assessment				First tier step assessment					
	Formula	Shortcut value		Trigger value	Formula	Exposure factor	twa	Shortcut value		Trigger value
		Down-ward	Side-ward					Down-ward	Side-ward	
Acute oral exposure	ETR=AR×SV/72-h $LD_{50\ oral}$	11.2	13.3	0.036	ETR=AR×Ef×SV /72-h $LD_{50\ oral}$	1	-	11.2	13.3	0.036
Chronic oral exposure	ETR=AR×SV/10-d $LDD_{50\ oral}$	11.2	13.3	0.0048	ETR=AR×Ef×SV ×twa/10-d $LDD_{50\ oral}$	1	0.72	9.9	11.4	0.0048

Table S2. Comparative analysis of survival rate of bumblebee workers exposed to different concentrations of flupyradifurone and solvent controls.

Species	Concentration (μg/mL)					
	15	30	60	120	240	480
Bombus terrestris	$\chi^2 = 0.0001$	$\chi^2 = 0.3605$	$\chi^2 = 2.9268$	$\chi^2 = 9.0823$	$\chi^2 = 18.9517$	$\chi^2 = 50.6785$
	$P = 0.9904$	$P = 0.5482$	$P = 0.0871$	$P = 0.0026$	$P < 0.0001$*	$P < 0.0001$*
	**Concentration (μg/mL)**					
	10	20	40	80	160	320
Bombus lantschouensis	$\chi^2 = 1.4747$	$\chi^2 = 3.3010$	$\chi^2 = 19.0974$	$\chi^2 = 47.5114$	$\chi^2 = 64.1131$	$\chi^2 = 66.2714$
	$P = 0.2246$	$P = 0.0692$	$P < 0.0001$*	$P < 0.0001$*	$P < 0.0001$*	$P < 0.0001$*

This analysis corresponds to Figure 3.
